# Sciatica leading to the discovery of a renal cell carcinoma

**DOI:** 10.4314/pamj.v9i1.71193

**Published:** 2011-06-10

**Authors:** Mohamed Amine Lakmichi, Redouane Jarir, Jamal Kabour, Zakaria Dahami, Mohamed Said Moudouni, Ismail Sarf

**Affiliations:** 1 Department of Urology, Faculty of Medicine and Pharmacy, University Hospital Center Mohammed the VIth, Cadi Ayyad University of Marrakesh, Morocco

**Keywords:** Renal carcinoma, kidney cancer, metastatic renal cell cancer, cytoreductive nephrectomy

## Abstract

Metastatic renal cell cancer is not exceptional in kidney cancer (30% of patients with kidneyl cancer). Its prognosis is particularly severe. However, sciatic neuralgia (sciatica) remains an exceptional revealing clinical sign of this disease. The authors report the case of a patient admitted with right sciatica as chief complain, leading to the discovery of a renal cell carcinoma. Although uncommon, renal cell carcinoma spine metastasis should be included in the differential diagnosis of back pain and sciatica.

## Introduction

Kidney cancer accounts for 3% of all malignancies in adults and is the third most common urological cancer after prostate and bladder cancers [1].The metastatic form is not uncommon (30% of patients with kidney cancer) and the prognosis is particularly severe. The most frequent secondary locations of kidney cancers are lung, bone, liver and brain. Many other sites have been described in the literature. Sciatica is a relatively rare mode of presentation of renal cell carcinoma. The authors report the case of a patient who consulted for sciatica associated with a mass of the right buttocks leading to the discovery of a metastatic renal cell carcinoma.

## Patient and case report

**Figure 1:**
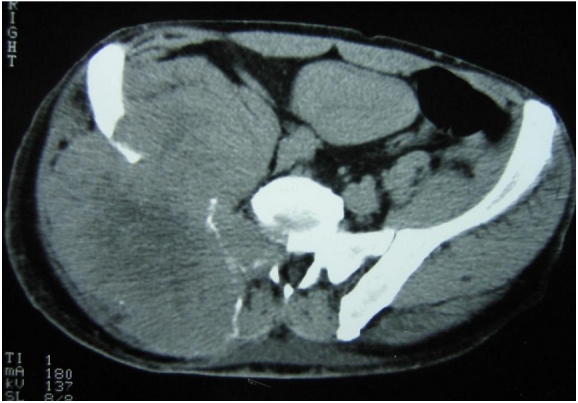
TDM showing a right buttocks and iliac fossa mass with destruction of adjacent structures and invasion of the spinal canal in a patient who consulted for sciatica leading to the discovery of a renal cell carcinoma

**Figure 2:**
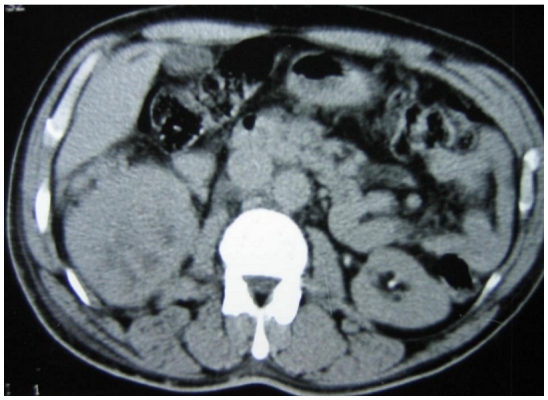
TDM showing a right kidney mass in a patient who consulted for sciatica leading to the discovery of a renal cell carcinoma

A 52-year-old male was admitted to the Neurosurgery Department for right leg sciatica with paraesthesia. Clinical examination found a patient in relatively good general conditions. However, a mass in the right buttock was noted. The mass was hard, tender but without signs of inflammation. There was significant decrease in muscle strength and reflexes of the right leg. A pelvic CT scan (CT) showed a mass of 19 cm in diameter rising from the sacroiliac region (bone and soft tissue). It had straight edge, heterogeneous tissue density with osteolysis in the body of the 11th thoracic vertebra and invasion of its spinal canal (Figure. 1). Abdominal CT showed a 10 cm right upper pole renal mass (Figure. 2).

Several biopsies of the gluteal mass were performed. Pathology confirmed a renal cell carcinoma (Fuhrman grade III). A cytoreductive nephrectomy associated to a postoperative immunotherapy was planned to our patient. An open radical cytoreductive nephrectomy was performed. Postoperative evolution was uneventful. Pathological examination later confirmed renal cell carcinoma. External radiotherapy for analgesia in order to improve the quality of life was performed. Postoperative immunotherapy could not be achieved. The patient died six months later, in the setting of cancer cachexia.

## Discussion

Kidney cancer accounts for 3% of all malignancies in adults and is the third most common urological cancer after prostate cancer and bladder cancer [1]. It is characterized by an unpredictable clinical course. The primary tumor location may be revealed by multiple metastases, or otherwise remain quiescent for years [2]. On diagnosis, over one third of patients are already in metastatic stage with multiple locations [3]. The metastatic disease is not uncommon and is usually associated with a more severe prognosis. The median survival of these patients is about 10 months.

Synchronous metastases (discovered during the identification of the primary lesion) in kidney cancer are often multiple. The most common metastatic sites of renal cancer are the lung (50 to 60% of cases), lymph nodes (15-30%), bone (30-40%), liver (28%), the adrenals (10-15%) and brain (10 to 13%). Kidney cancer, specifically clear cell carcinoma can metastasize to all organs [4]. Unusual sites can be achieved such as the gastrointestinal tract, genital organs, retro-peritoneum, muscles, skin, heart, breast, head and neck, etc. [4]. Renal metastases have been described in skeletal muscle, with a relatively low incidence on autopsy series (0 to 1.6%). There is no preferential site muscle, any muscle can be invaded (masseter, quadriceps, trapezius, biceps, etc.). Often, these metastases were discovered well after the kidney tumor (10 months to 16 years) [5]. Among those metastatic sites, a clinical presentation with sciatica was an uncommon event.

There are two main routes for the metastatic spread of kidney cancer: the blood (haematogenous) and lymphatic route. The most frequent route is haematogenous, leading to the lungs via the renal vein, vena cava and right atrium. This mode of spread cells was observed for pancreatic metastases, spleen, intestine and heart, which are, therefore, often preceded by pulmonary metastases [2].

Atypical renal metastasis can be explained by arterial microemboli, but there are some more specific ways of disseminating. Thus, metastases to the testicle, spermatic cord, vagina and ovary can be explained by a retrograde spread from the gonadal vein. The existence of a renal vein thrombus (left) or inferior vena cava (right), promote the spin-cell tumor in the spermatic vein reflux, as well as the occurrence of varicocele. [6]. Time between metastasis and primary tumor varies different depending on the type of organ affected. For example, pancreatic metastases appear on average 10 years after the primary tumor and ranged from 1 month to 27 years [7] while intra-thyroid metastases have an average time of onset of 6 to 10 years [8]. These metastases can be found at diagnosis (synchronous metastases) or, more rarely, precede the renal tumor in 5% of cases [9]. Some locations such as skin, intestines, heart are rarely isolated and are often preceded by other secondary sites. Sometimes metastatic patterns is revealed by a paraneoplastic syndrome (anemia, thrombocytopenia, etc.) [10].

When clinical symptoms and imaging data have revealed the secondary location, say its renal origin is sometimes difficult. It is then possible to make use of a thin biopsy needle to characterize the origin of the tumor as it was done in our patient.

Metastatic kidney cancer by definition has a poor prognosis with survival rates at 5 years ranging from 7 to 13%. The management of synchronous metastases raises several questions concerning the primary role of nephrectomy. The character of synchronous metastases is a factor of poor prognosis, compared with asynchronous metastases. Most studies concerned with cytoreductive nephrectomy in patients with metastatic disease are retrospective with small patient samples. In these studies, the operative mortality ranged between 0% and 17% with an operative morbidity ranging from 13% to 50% and a number of patients unable to receive systemic therapy between 7% and 77% [11]. Our patient was in this setting unable to receive immunotherapy. However, the most important argument in favor of cytoreductive nephrectomy in this setting was that reported by a prospective, randomized study of the SWOG (Southwest Oncology Group) and EORTC (European Organization for Research and Treatment of The Cancer) [12]. They reported a median survival of 13.6 months for nephrectomy plus interferon group vs 7.8 months for interferon alone group [12]. But these studies did not report whether cytoreductive nephrectomy should be performed before or after systemic therapy. It is now clear that cytoreductive nephrectomy in patients with metastatic disease with a good performance status and easily resectable tumor is a reasonable option. The tumor in our case was perfectly résécable allowing us to perform the nephrectomy in good conditions especially with the good performance status of our patient. Nevertheless, selection of patients for this procedure should be judicious having in mind that this gesture is not curative [13].

Arguments to justify nephrectomy exist; it could lead to spontaneous regression of metastases, it would prevent the onset of symptoms due to the primary lesion, it would allow the correction of para-neoplastic syndromes and finally it would maximize the impact of immunotherapy. Nevertheless, some arguments against also exist, leading to not recommend nephrectomy: patients are often delayed for immunotherapy, surgery having a deleterious effect by inducing a specific immune deficiency [14]. However, it remains important to note that the level I evidence that supports cytoreductive nephrectomy is only for patients who will be candidates for adjuvant immunotherapy. Moreover, similar studies do not yet exist for targeted therapies [13]. Our patient was scheduled for an adjuvant immunotherapy. However, his general conditions began to deteriorate leading to analgesic radiotherapy to relieve him.

The advantage of laparoscopic nephrectomy in this setting was reported in the literature [15]. It has the demonstrated advantage of reducing perioperative morbidity and therefore significantly reduces the time between the intervention and the start of systemic therapy. However, this technique currently remains conditional on an appropriate selection of patients. Several prognostic factors influence patient survival: age, general health, weight loss, time to metastasis from the primary tumor and the number of metastatic sites [16].

## Conclusion

Metastatic kidney cancer, which has a very negative prognosis, is currently enjoying some interesting developments. New medical approaches (targeted therapies) appear promising for the future. However, further studies including those assessing therapeutic options in large samples of patients are needed.
